# Glycine and Folate Ameliorate Models of Congenital Sideroblastic Anemia

**DOI:** 10.1371/journal.pgen.1005783

**Published:** 2016-01-28

**Authors:** J. Pedro Fernández-Murray, Sergey V. Prykhozhij, J. Noelia Dufay, Shelby L. Steele, Daniel Gaston, Gheyath K. Nasrallah, Andrew J. Coombs, Robert S. Liwski, Conrad V. Fernandez, Jason N. Berman, Christopher R. McMaster

**Affiliations:** 1 Department of Pharmacology, Dalhousie University, Halifax, Canada; 2 Department of Pediatrics, IWK Health Centre, Dalhousie University, Halifax, Canada; 3 Department of Pathology, Dalhousie University, Halifax, Canada; 4 Qatar University, Doha, Qatar; Stanford University School of Medicine, UNITED STATES

## Abstract

Sideroblastic anemias are acquired or inherited anemias that result in a decreased ability to synthesize hemoglobin in red blood cells and result in the presence of iron deposits in the mitochondria of red blood cell precursors. A common subtype of congenital sideroblastic anemia is due to autosomal recessive mutations in the *SLC25A38* gene. The current treatment for *SLC25A38* congenital sideroblastic anemia is chronic blood transfusion coupled with iron chelation. The function of *SLC25A38* is not known. Here we report that the SLC25A38 protein, and its yeast homolog Hem25, are mitochondrial glycine transporters required for the initiation of heme synthesis. To do so, we took advantage of the fact that mitochondrial glycine has several roles beyond the synthesis of heme, including the synthesis of folate derivatives through the glycine cleavage system. The data were consistent with Hem25 not being the sole mitochondrial glycine importer, and we identify a second SLC25 family member Ymc1, as a potential secondary mitochondrial glycine importer. Based on these findings, we observed that high levels of exogenous glycine, or 5-aminolevulinic acid (5-Ala) a metabolite downstream of Hem25 in heme biosynthetic pathway, were able to restore heme levels to normal in yeast cells lacking Hem25 function. While neither glycine nor 5-Ala could ameliorate *SLC25A38* congenital sideroblastic anemia in a zebrafish model, we determined that the addition of folate with glycine was able to restore hemoglobin levels. This difference is likely due to the fact that yeast can synthesize folate, whereas in zebrafish folate is an essential vitamin that must be obtained exogenously. Given the tolerability of glycine and folate in humans, this study points to a potential novel treatment for *SLC25A38* congenital sideroblastic anemia.

## Author Summary

Mutations in the *SLC25A38* gene cause an inherited anemia. In this study we determine that the function of SLC25A38, and its yeast homolgue Hem25, is to act as mitochondrial glycine importers providing a molecular explanation for why patients with *SLC25A38* mutations have low hemoglobin levels and become anemic. Using this new knowledge, we go on to determine that supplementation with glycine and folate restore hemoglobin levels in a zebrafish model of the disease pointing to a potentially new, safe, and cost effective treatment for *SLC25A38* congenital sideroblastic anemia.

## Introduction

Sideroblastic anemias are a group of disorders principally defined by a decreased level of hemoglobin in erythrocytes (red blood cells) and the presence of pathological iron deposits in perinuclear mitochondria of erythroblasts (red blood cell precursors found in bone marrow) [1–5]. Sideroblastic anemias can be congenital or acquired with both primarily being due to a defect in heme/hemoglobin synthesis. One of the main reasons for acquired sideroblastic anemia is a nutritional deficiency in vitamin B6 (pyridoxine) as several of the enzymes required to synthesize heme and heme precursors require pyridoxal 5’-phosphate (PLP) as a cofactor. Alcohol abuse, copper deficiency, lead poisoning, some antimicrobial drugs, and myelodysplastic syndrome can also result in acquired sideroblastic anemia. Mutations in several genes cause congenital sideroblastic anemia (CSA) including *ALAS2*, *SLC25A38*, *ABCB7*, *GLRX5*, *SLC19A2*, *PUS1*, and *YAR2*.

The two most common types of CSA are an X-linked form due to mutations in *ALAS2* and the more recently identified autosomal recessive form due to mutations in *SLC25A38* [6–9]. *ALAS2* and *SLC25A38* are primarily expressed in erythroid precursor and red blood cells. ALAS2 is a PLP-dependent enzyme that catalyzes the first enzymatic step of the heme/hemoglobin biosynthesis pathway utilizing glycine and succinyl-CoA to synthesize 5-aminolevulinic acid (5-Ala). A subset of *ALAS2* CSA patients, those with mutations that decrease PLP binding, can be treated with high levels of pyridoxine. *ALAS2* CSA patients with mutations outside of the PLP binding region, and all *SLC25A28* CSA patients, are refractory to pyridoxine treatment.

Pyridoxine refractory CSA patients suffer severe clinical consequences including a microcytic transfusion-dependent anemia that usually appears in infancy resulting in sequelae typical of chronic transfusion therapy, and can suffer significant long term morbidity and mortality related to iron overload [10]. Recently, the adoption of effective and tolerable oral iron chelation therapies predict an increase in life expectancy comparable to that found for transfusion-dependent adequately chelated patients with hemoglobinopathies [11]. Despite these advances, oral iron chelators carry their own risks [12] and lifetime transfusion is associated with high financial quality of life burdens, with additional medical complications including alloimmunization and acquired infectious agent transmission including hepatitis B and C [13]. There is a clear need to decrease transfusion dependence for CSA patients. Here, we employ yeast and zebrafish as complementary preclinical models to determine the function of SLC25A38 and go on to propose a potential therapy for *SLC25A38* CSA patients.

## Results

### Determining the Function of SLC25A38

**Fig 1:**
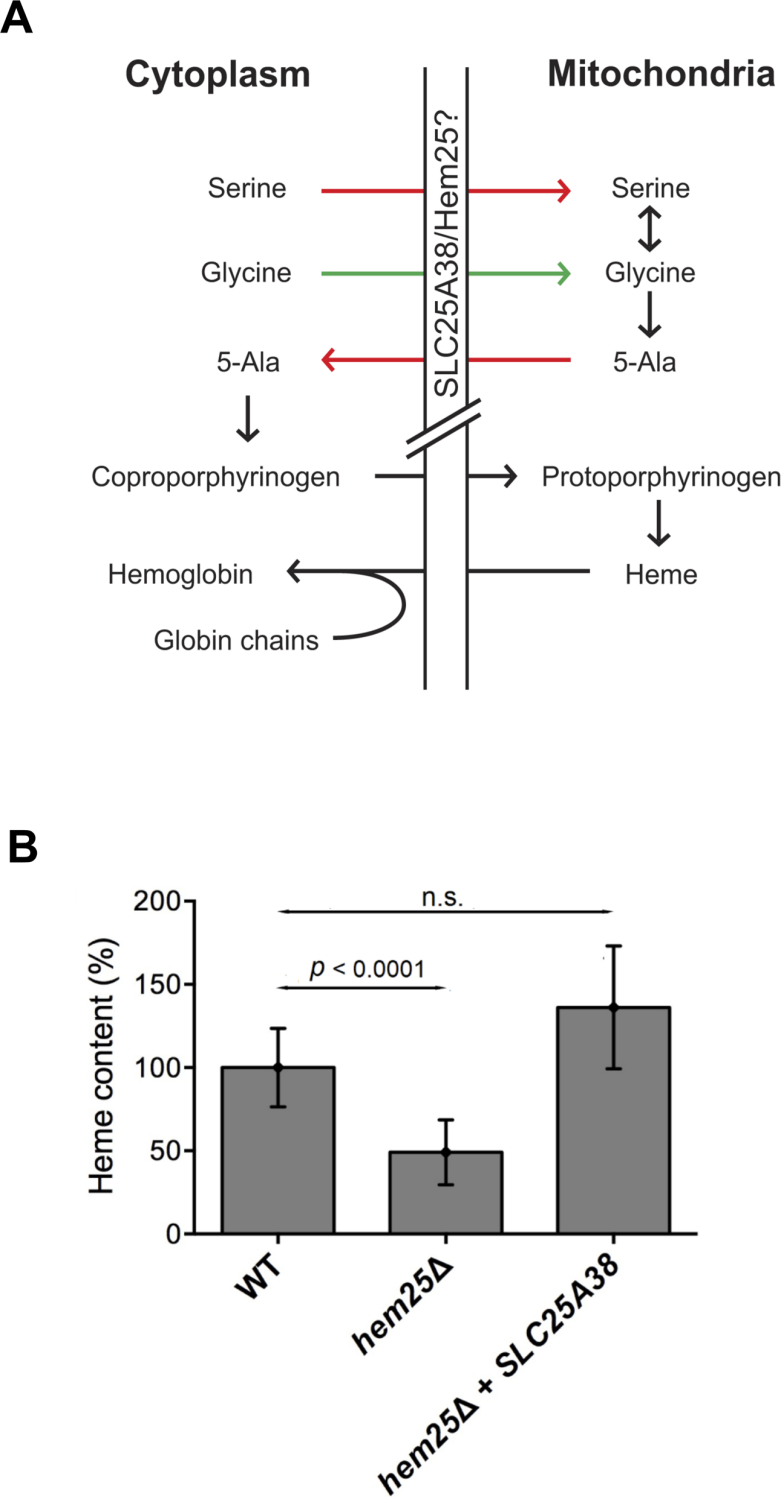
*SLC25A38* encodes a mitochondrial glycine importer. A) Illustration of the potential roles for SLC25A38 in the synthesis of heme. SLC25A38 has been postulated to act as a glycine or serine importer for the subsequent synthesis of heme/hemoglobin. B) Yeast cells of the indicated genotypes were grown to mid-log phase and cells were processed for heme determination. Wild type cell heme content was 31.1 fmol/μg protein. Heme values are the mean ± SEM of at least six independent determinations with *p* values determined using t-test.

The function of SLC25A38 is not known (Fig 1A). To determine how *SLC25A38* mutations cause CSA we sought to determine its function using a yeast model. The *Saccharomyces cerevisiae SLC25A38* homologue *YDL119c*, which we name *HEM25* (*Hem*e synthesis by SLC*25* family member), was inactivated in the yeast genome and the level of heme was determined. The *hem25*Δ yeast cells exhibited a 50% decrease in the level of heme (Fig 1B). Human *SLC25A38* was expressed in *hem25*Δ cells in order to determine if the human protein could complement the absence of the yeast protein. Heme level was restored to normal upon expression of the human SLC25A38 protein in yeast cells with an inactivated *HEM25* gene indicating conservation of function between the yeast and human proteins. SLC25A38 is a member of the mitochondrial SLC25 family which are subdivided into keto acid, adenine nucleotide, and amino acid carriers [14]. Phylogenetic analysis of human SLC25A38 grouped SLC25A38 with the amino acid carriers (S1 Fig). This is consistent with previous predictions that SLC25A38 could be a mitochondrial glycine or serine transporter required for the synthesis of heme [6].

**Fig 2:**
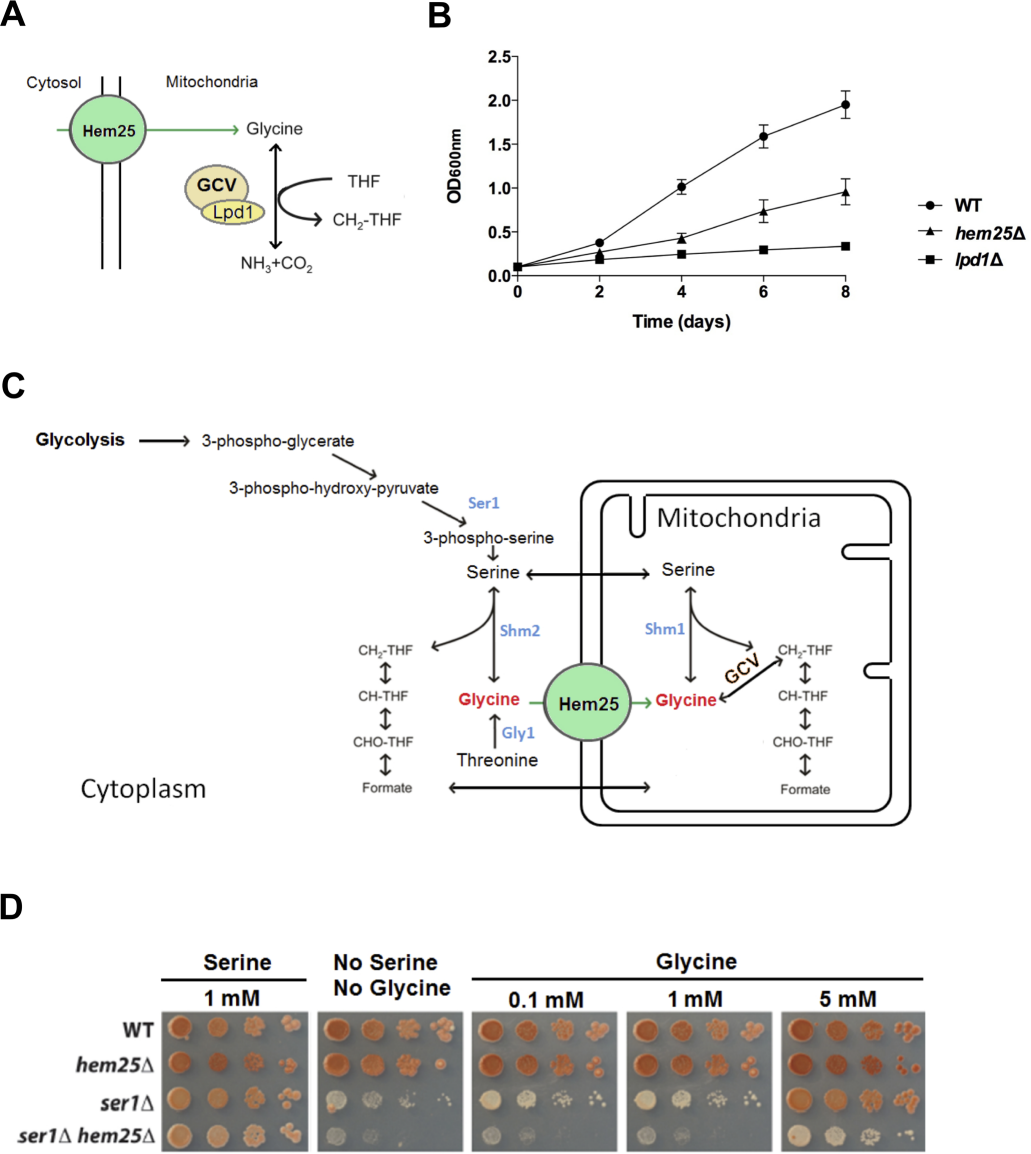
Hem25 is required for the effective import of glycine into mitochondria. A) Glycine can serve as the sole nitrogen source in *S*. *cerevisiae*. The GCV converts glycine to NH_3_, as the GCV resides in the mitochondria the use of glycine as a nitrogen source requires efficient uptake of glycine into the mitochondria. B) Inactivation of the *HEM25* gene in yeast substantially decreased their ability to grow on glycine as the sole nitrogen source. Cells were grown in SD medium containing 30 g/l glycine as nitrogen source. Growth was determined by optical density (OD) of the culture at 600 nm. Data shown are the mean ± SEM for four replicates for wild type and *lpd1*Δ cells and nine replicates for *hem25*Δ cells. C) Serine is synthesized from the glycolytic intermediate 3-phosphoglycerate through a series of reactions that includes phosphoserine transaminase (PSAT1 in humans, Ser1 in *S*. *cerevisiae*). Serine is normally the main source of one carbon units (CH_2_-THF, 5,10 methylenetetrahydrofolate and its metabolites) in cells. Inactivation of the *SER1* gene in yeast results in yeast cells that are auxotrophic for serine. Glycine supplementation can also overcome a mutation in the *SER1* gene as glycine can serve as a metabolic source for both serine and one carbon units. However, this capacity depends entirely on mitochondrial glycine import. The import of glycine into the mitochondria can generate one carbon units in the form of CH_2_-THF through the activity of the glycine cleavage system (GCV). In addition, mitochondrial serine hydroxymethyltransferase (SHMT2 in humans, Shm1 in yeast) catalyzes the synthesis of serine from glycine and CH_2_-THF, with serine exported into the cytoplasm to be consumed for several anabolic pathways including the synthesis of CH_2_-THF. Simultaneously, CH_2_-THF generated from glycine is oxidized to formate and also exported into the cytoplasm as a source of cytoplasmic one carbon units. D) An inability to import glycine into the mitochondria prevents glycine supplementation from providing serine and one carbon units to cells with an inactivated *SER1* gene. This was found to be the case upon inactivation of the yeast *HEM25* gene. Cells were grown to mid-log phase in SD medium containing 1 mM serine, and 1:10 serial dilutions plated on SD medium with no supplements or supplemented with serine or glycine.

We first sought to determine if Hem25 is a mitochondrial glycine importer. To do so, we used two separate scenarios where the efficient uptake of glycine by mitochondria was required for yeast cell growth. In the first scenario, we took advantage of the fact that glycine can be used as the sole nitrogen source by yeast cells, but only if glycine is efficiently imported into mitochondria for conversion to NH_3_ by the glycine cleavage system (GCV) (Fig 2A). The ability of *hem25*Δ cells to grow with glycine as the sole source of nitrogen was determined. Inactivation of the *HEM25* gene impaired the ability of cells to grow when glycine was the sole nitrogen source, although not to the extent observed for inactivation of an enzyme of the GCV, dihydrolipoamide dehydrogenase encoded by *LPD1* (Fig 2B). The results are consistent with Hem25 being an important importer of glycine into mitochondria.

In the second scenario, we used the observation that in yeast cells with an inactivated *SER1* gene [15] glycine supports the growth of *ser1*Δ cells but only if glycine can be efficiently imported into the mitochrondria. In this context, glycine becomes the major source of one carbon units via its catabolism by the mitocondrial glycine cleavage system (GCV) to produce CH_2_-THF, and of serine through mitochondrial serine hydroxymethyltransferase which itself requires CH_2_-THF (Fig 2C) [16–19]. To determine if Hem25 is required for effective glycine import into mitchondria, the *HEM25* gene was inactivated in a *ser1*Δ background and the ability of *ser1*Δ and *ser1*Δ *hem25*Δ strains to grow in the presence of serine or glycine was determined. As expected, serine supported the growth of all strains (Fig 2D). Congruent with the dependence on GCV for the generation of one carbon units and subsequent serine synthesis, *ser1*Δcells grew poorly on non-supplemented medium. Importantly, the absence of Hem25 in *ser1*Δ cells exacerbated the growth defect. Glycine supported growth of *ser1*Δ cells, however, *ser1*Δ *hem25*Δ cells grew poorly in medium containing low concentrations of glycine (Fig 2D), consistent with Hem25 being required to efficiently import glycine into mitochondria. Interestingly, as the levels of glycine were titrated upward, there was an increase in growth of the *ser1*Δ *hem25*Δ cells, implying other mitochondrial glycine transporter(s) (of lower glycine affinity) may exist.

**Fig 3:**
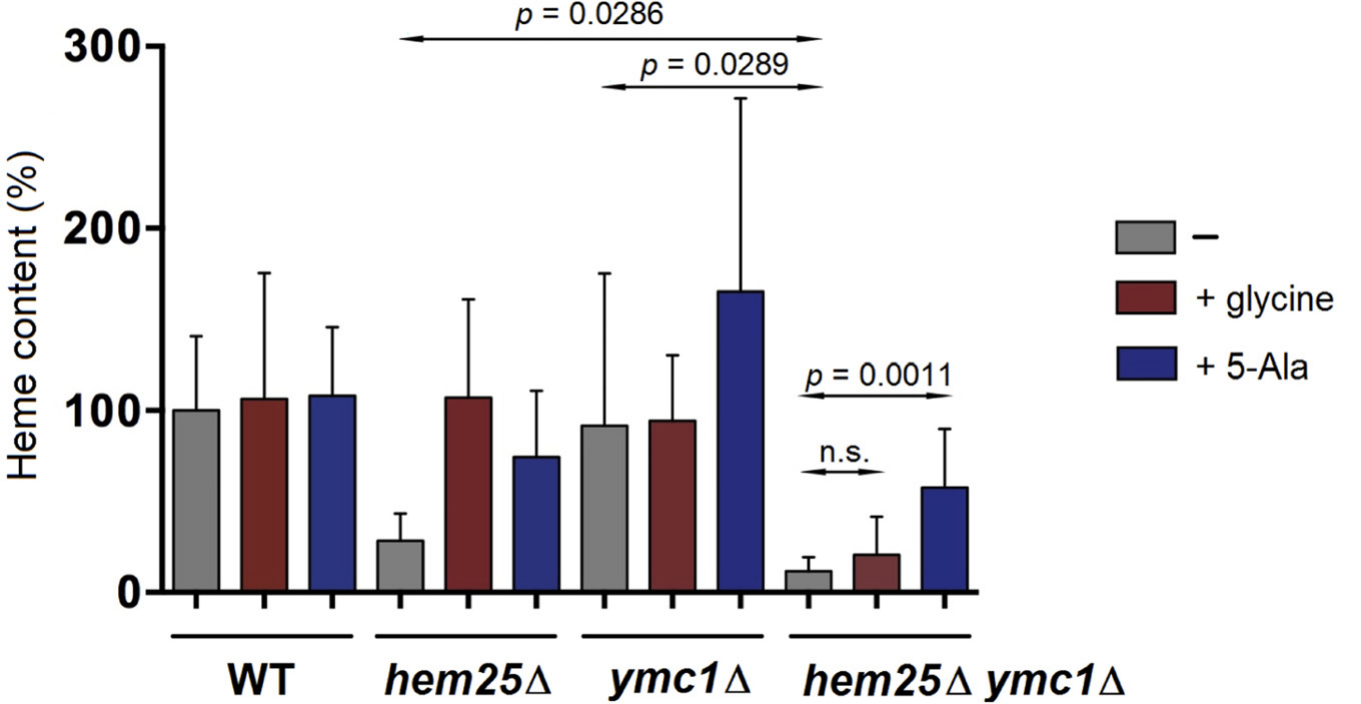
Identification of a putative second mitochondrial glycine importer. Yeast cells of the indicated genotypes were grown to mid-log phase in the presence of no supplements or the addition of 5 mM glycine or 50 μg/ml 5-Ala. Cells were processed for heme determination.

To determine if other members of the yeast SLC25 family member could be secondary glycine transporters, each gene encoding a putative amino acid carrier that was a member of the SLC25 family was inactivated simultaneously with the *HEM25* gene. The ability of each single and double mutant to synthesize heme in the presence of glycine or 5-Ala was determined. If any of these SLC25 family members encode a secondary glycine transporter, then 5-Ala, but not high levels of glycine should restore heme levels to *hem25*Δ cells also lacking a specific SLC25 family member. This was observed for *hem25*Δ *ymc1*Δ cells. The double *hem25*Δ *ymc1*Δ mutants had a significant reduction in heme content compared with the single *ymc1*Δ or *hem25*Δ mutants (Fig 3), consistent with both genes being on parallel pathways contributing to the same downstream product, in this case heme. Double mutant cells grown on media supplemented with 5 mM glycine did not show an increase in heme content whereas double mutant cells supplemented with 5-Ala showed an increase in heme content, although not to wild type levels.

The data strongly support the notion that SLC25A38/Hem25 is a glycine transporter required to synthesize heme. However, it is also clear that in yeast Hem25 is not the sole mitochondrial glycine transporter and secondary glycine transporter(s) exist. We identify Ymc1 as a putative secondary mitochondrial glycine transporter that contributes to heme synthesis when glycine concentrations are high.

### Cytoplasmic Threonine Aldolase Is the Main Source of *De Novo* Synthesized Glycine Used for Heme Synthesis

**Fig 4:**
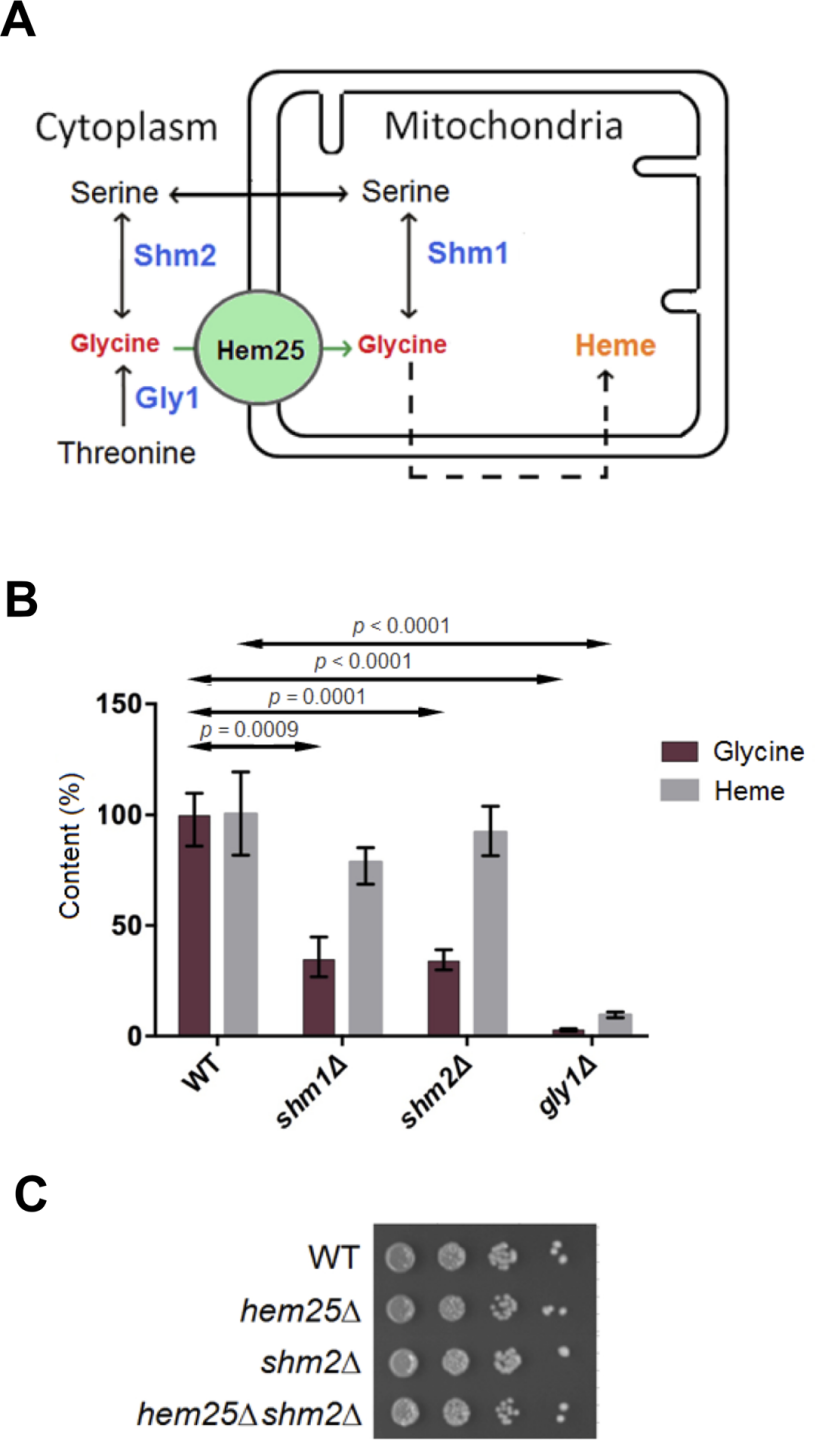
Cytosolic threonine aldolase is the main soruce of glycine for heme synthesis. A) Glycine can be synthesized in glucose grown yeast via three enzymes, Gly1 (threonine aldolase), and cytosolic (Shm2) and mitochondrial (Shm1) serine hydroxymethyltransferases. B) Yeast cells of the indicated genotypes were grown to mid-log phase and cells were processed for glycine and heme determination. Wild type glycine was 6.0 nmol/10^8^ cells. C) Cells of the indicated genotypes were grown to mid-log phase and 1:10 serial dilutions plated on SD medium with no supplements.

We next sought to determine the *de novo* source(s) of glycine used for heme synthesis. The genes encoding enzymes that synthesize glycine under glucose grown conditions are *GLY1* encoding a cytoplasmic threonine aldolase, *SHM2* encoding a cytoplasmic serine hydroxymethyltransferase, and *SHM1* encoding a mitochondrial serine hydroxymethyltransferase (Fig 4A). The *GLY1*, *SHM1* and *SHM2* genes were inactivated and the levels of glycine and heme determined in *gly1*Δ, *shm2*Δ, and *shm1*Δ single mutant cells. Inactivation of the *GLY1* gene, encoding cytoplasmic threonine aldolase, reduced cellular heme by 75% and glycine mass by 90% (Fig 4B). Upon inactivation of either the cytoplasmic or mitochondrial serine hydroxymethyltransferases, a 50% decrease in glycine was observed, however, the level of heme level was not significantly different from that of wild type cells. As Gly1 produces glycine in the cytoplasm, this is consistent with a cytoplasmic source of glycine being the main contributor to heme synthesis through its import by the mitochondrial SLC25A38/Hem25 glycine transporters for subsequent use as substrate in the first enzymatic step in the synthesis of heme catalyzed by ALAS2/Hem1.

### Hem25 Is Not a Serine Transporter

A second hypothesis for the function of SLC25A38/Hem25 was as a serine transporter. This was based on the metabolic pathway whereby serine, once imported into the mitochondria, can be converted to glycine by mitochondrial serine hydroxymethyltransferase (Shm1) for subsequent use by ALAS2 (Hem1 in yeast) to initiate heme synthesis (Figs 1A and 4A). If this was the case, then loss of function of Shm1 should lead to a significant decrease in the level of heme, however, in *shm1*Δ cells heme level was similar to wild type (Fig 4B), as opposed to the profound effect on heme level observed in *hem25*Δ cells (Fig 1B).

To further investigate if Hem25 could be a serine transporter, we took advantage of the fact that it has been reported that *shm1*Δ cells grow with rates similar to wild type cells whereas *shm2*Δ cells exhibit a growth impairment associated with a shortage of one-carbon units that can be alleviated by formate supplementation (formate is transported between the mitochondria and cytoplasm as part of the folate cycle (Fig 2C) [15]. Consistent with this observation, there are two studies that showed that *shm2*Δ *shm1*Δ cells have impaired growth compared to *shm2*Δ cells [15,20] with the addition of exogenous formate restoring growth to wild type level. If Hem25 were a serine transporter, the growth phenotype of the *shm2*Δ *hem25*Δ cells should be worse than *shm2*Δ cells as the absence of Hem25 would prevent the entry of serine into the mitochondria and restrict the generation of one-carbon units by Shm1. However, there was no difference in growth of *shm2*Δ versus *shm2*Δ *hem25*Δ cells (Fig 4C). As the absence of Hem25 does not aggravate the growth phenotype of *shm2*Δ cells this suggests that Hem25 is not a serine transporter.

The third experiment we performed to determine if Hem25 was a serine importer was based on the observation of McNeil [15] *et al* that reported that *shm2*Δ *gcv1*Δ cells had a slight growth defect, which was dramatically worsened by inactivation of *SHM1*. Both strains had their growth rates restored to wild type by formate addition. If Hem25 was involved in serine import, *shm2*Δ *gcv1*Δ *hem25*Δ cells should grow slower compared to *shm2*Δ *gcv1*Δ cells as the absence of Hem25 would deprive Shm1 of its substrate serine, limiting the generation of one-carbon units. We determined if there was a difference in growth for *shm2*Δ *gcv1*Δ isolates and *shm2*Δ *gcv1*Δ *hem25*Δ strains and observed that the double and triple mutants strains grew at similar rates (S2 Fig), again indicating that Hem25 does significantly not contribute to the import of serine into the mitochondria for its subsequent conversion to glycine by Shm1.

### Glycine and 5-Ala Rescue the Heme Biosynthetic Defect in the Yeast Model of CSA

**Fig 5:**
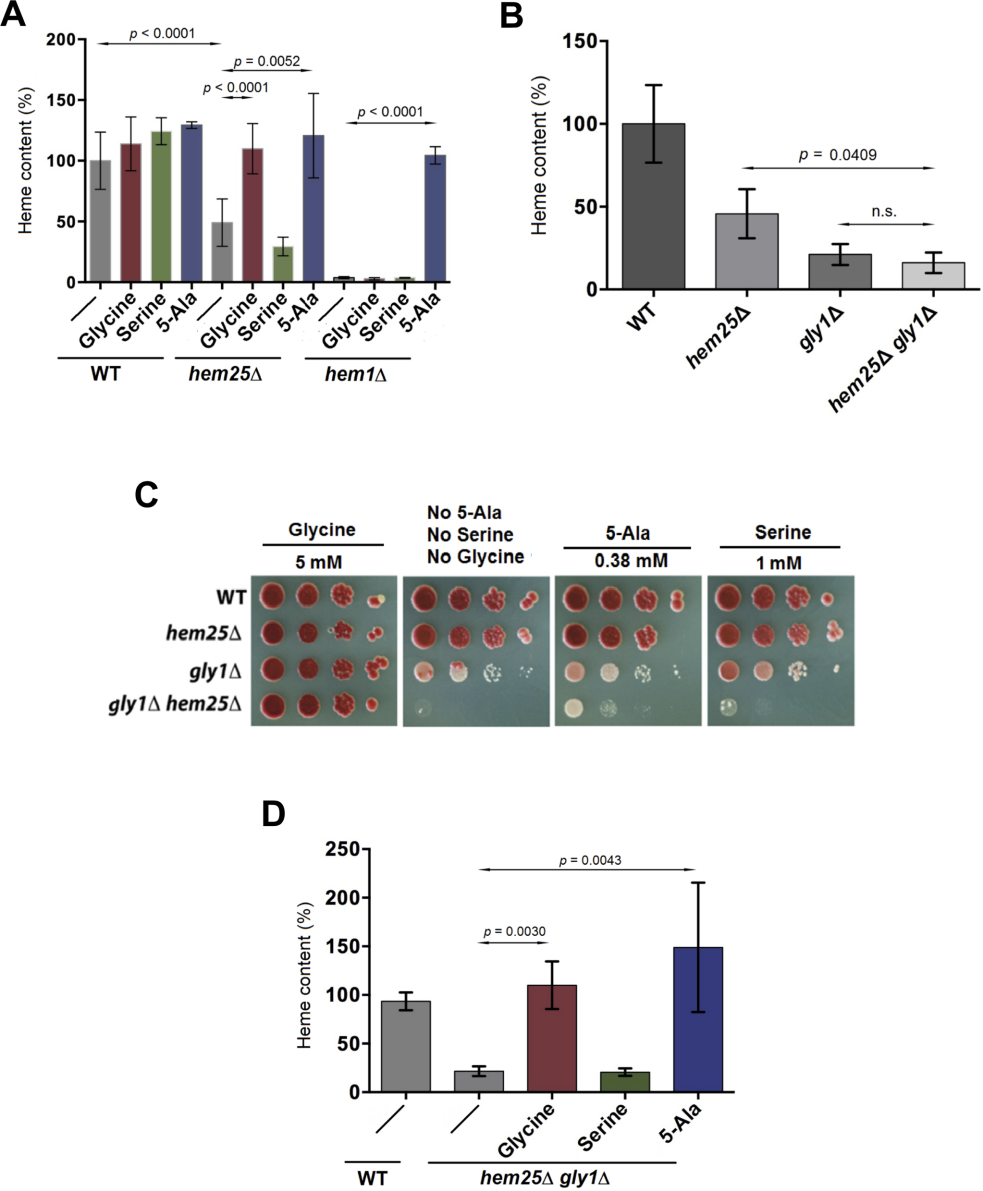
Rescue of growth and heme content in yeast models of congentical sideroblastic anemia. A) Yeast cells were grown to mid-log phase in the absence or presence of 5 mM glycine, 1 mM serine or 50 μg/ml 5-Ala (the *hem1*Δ strain is normally grown in the presence of 0.5 μg/ml 5-Ala to allow for growth at a wild type rate) and cells were processed for heme determination. Heme values are the mean ± SEM of at least six independent determinations. B) Yeast cells of the indicated genotypes were grown to mid-log phase and processed for heme determination. C) Cells were grown to mid-log phase in SD medium containing 1 mM serine, and 1:10 serial dilutions plated on SD medium supplemented with glycine, 5-Ala, or serine. D) Cells were grown to mid-log phase in the absence or presence of 5 mM glycine, 1 mM serine or 50 μg/ml 5-Ala and processed for heme determination.

We hypothesized three scenarios to ameliorate the defect in heme synthesis in *hem25*Δ cells (i) supplementation with exogenous glycine to increase substrate availability for the first step in heme synthesis, (ii) addition of excess serine to drive endogenous glycine synthesis in mitochondria, or (iii) addition of the downstream metabolite, 5-Ala, within the heme biosynthesis pathway. Each compound was added to wild type, *hem25*Δ, and *hem1*Δ cells. Serine had no effect on the growth of any of these strains. The addition of 5-Ala restored heme to normal levels in *hem1*Δ and *hem25*Δ cells, while only the addition of exogenous glycine restored heme level to normal in *hem25*Δ cells (Fig 5A). These latter two results are consistent with Hem25 lying upstream of Hem1 in the synthesis of heme. We also determined the level of heme in the *hem25*Δ *gly1*Δ cells and observed that it was not substantially diminished beyond that observed in *gly1*Δ cells alone (Fig 5B), consistent with two proteins on a linear pathway synthesizing the same downstream product.

Interestingly, we noted a growth defect for *gly1*Δ and *hem25*Δ *gly1*Δ cells that could be differentially restored by the addition of 5-Ala (Fig 5C). For the single *gly1*Δ single mutant, 5-Ala did not increase growth whereas glycine restored growth to wild type levels. Thus, although the level of heme is depleted in *gly1*Δ cells its level does not limit cell growth, as 5-Ala does not restore growth. Some other glycine dependent process must be limiting *gly1*Δ cell growth when heme levels are normal. In the absence of supplements, the growth of the *hem25*Δ *gly1*Δ double mutant was slower than either single mutant alone. The addition of glycine or 5-Ala to the *hem25*Δ *gly1*Δ double mutant restored heme to normal levels (Fig 5D). Similar to *gly1*Δ cells, 5-Ala supplementation partially restored cell growth while glycine completely restored cell growth, indicating that growth impairment of the double mutant is only partly due to a decrease in heme level.

In the absence of exogenous glycine, cytoplasmic Gly1 is the major supplier of glycine for the synthesis of heme, consistent with the requirement for a high affinity mitochondrial glycine transporter being required for heme synthesis. When Gly1 function is inactivated glycine becomes limiting for cellular functions resulting in impaired growth, with simultaneous loss of Hem25 function resulting in a more severe growth defect. The growth defect of *gly1*Δ and *hem25*Δ *gly1*Δ cells cannot be restored through restoration of heme synthesis alone through the addition of 5-Ala, but can be restored by the addition of glycine, implying other glycine dependent functions are what impair cell growth.

### Rescue of the Zebrafish Model of CSA

**Fig 6:**
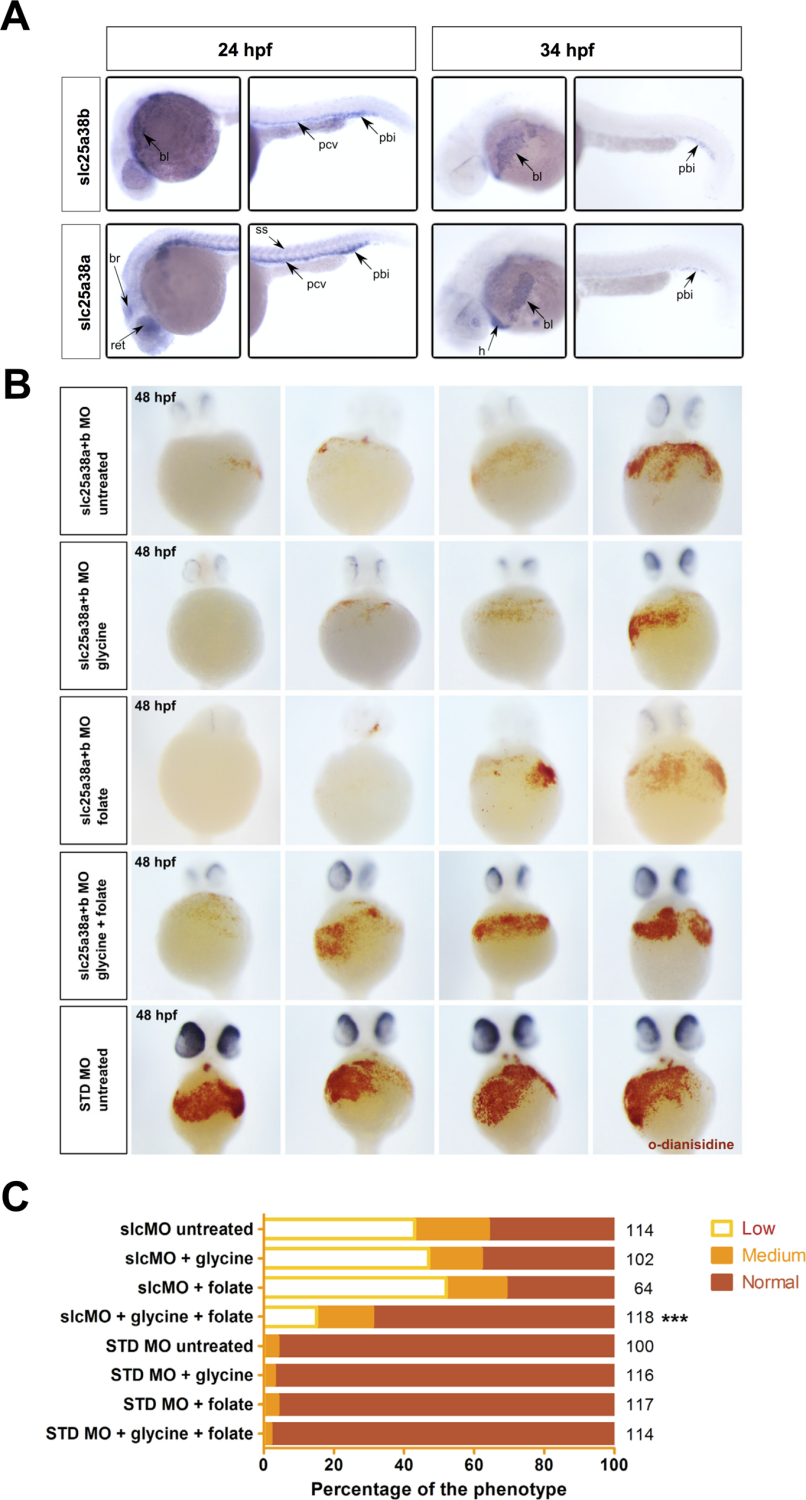
Rescue of the zebrafish model of congenital sideroblastic anemia. A) Whole-mount *in situ* hybridizations with probes for *slc25a38a* and *slc25a38b* at 24 and 34 hpf stages of development demonstrate predominant erythroid expression. For each stage and probe, head and tail views are shown and sites of expression are labeled. Abbreviations: pbi–posterior blood island, pcv–posterior cardinal vein, ss–somites, bl–blood, ret–retina, br–brain, h–heart. Expression of *slc25a38b* was observed in the posterior blood island, blood and posterior cardinal vein consistent with its preferential expression in erythroid progenitors and erythrocytes, whereas *slc25a38a* had the same blood-related expression pattern features and was also expressed in retina, brain and somites at 24 hpf. B) Representative images for hemoglobin staining using *o*-dianisidine of 48 hpf zebrafish embryos injected with *slc25a38a+b* morpholinos, or standard control morpholino (STD MO), treated with 100 mM glycine, 1 mM sodium folate, or both, starting from 4 hours post-injection. To control for morpholino specificity, we also injected 5-mismatch (5MM) versions of the same *slc25a38a+b* morpholinos and stained the injected embryos with o-dianisidine. For STD MO and 5MM morphants, images from only the STD MO untreated group are presented because of the low variation in *o*-dianisidine scores of untreated embryos versus those where glycine and/or folate were present. C) Graph of the *o*-dianisidine staining heme scores of 48 hpf zebrafish embryos injected with either *slc25a38a+b* morpholinos (slcMO) or STD MO and then treated from 4 through to 48 hpf with glycine, folate, or both glycine plus folate. All embryos were scored as having “low”, “medium” or “normal” hemoglobin levels in a blinded manner based on visual inspection of *o*-dianisidine staining. The total numbers (right axis) of scored embryos are indicated, from a total of three independent experiments. The “***” indicates that the *p*-value between the numbers of embryos in different scoring categories for *slc25a38a+b* morphants treated with glycine and folate versus the untreated *slc25a38a+b* morphant group is <0.001.

The zebrafish homologue of *SLC25A38* is duplicated in the zebrafish genome, like many zebrafish genes. To further understand the loss-of-function phenotypes of both *slc25a38a* and *slc25a38b*, we studied their expression by *in situ* hybridization at 24 and 34 hrs post-fertilization (hpf), time points at which each of the two waves of definitive hematopoiesis occur in zebrafish [21]. At 24 hpf, *slc25a38b* was expressed predominantly in the posterior blood island, posterior cardinal vein and circulating blood. By contrast, *slc25a38a* was also expressed in somites, brain and retina at 24 hpf (Fig 6A and S3 Fig). At 34 hpf expression patterns of both genes became more strongly restricted to the posterior blood island and circulating blood cells.

Morpholino knockdown of both *slc25a38a* and *slc25a38b* was required to produce an anemia in zebrafish embryos as determined using *o*-dianisidine staining to assess hemoglobin content (Fig 6B and [6]). Specificity of the morpholino oligos used was controlled by using five mismatch versions, as well as a control morpholino, whose injection resulted in normal phenotypes of the resulting morphants (Fig 6B). We observed a 50% decrease in hemoglobin level in *slc25a38a+b* morphants and larger more immature appearing cells with less compact nuclei. This was despite the absence of pathological sideroblasts in erythroid cells (S4 Fig). This finding is in keeping with other zebrafish models of CSA, such as the mutant *sauternes* (*sau*), which results in loss-of-function of the zebrafish orthologue of *ALAS2* [22]. The absence of ringed siderocytes in the zebrafish may be a result of the early time point in embryogenesis in which the cells are analyzed without sufficient time for either endogenous iron overload, or excess iron that accumulates from transfusion therapy in patients.

We next determined if the addition of glycine or 5-Ala could rescue the capacity of *slc25a38a+b*, or *alas2* knockdown fish, to synthesize heme. Optimization of glycine and 5-Ala dosing was performed in wild type zebrafish embryos by toxicity studies evaluating the frequency of death and developmental abnormalities relative to the dose of drug, a dose of ~50% of the maximum tolerated dose was chosen for treatments (S1 Table). Glycine or 5-Ala was added to the egg water of *slc25a38a+b* or *alas2* morphants, and *o*-dianisidine staining was used to assess hemoglobin content. Interestingly, neither glycine nor 5-Ala was able to restore hemoglobin level in *alas2* or *slc25a38a+b* morphants (Fig 6B and 6C and S5 Fig).

We know from our work with yeast lacking Hem25 function that decreasing the *de novo* capacity to synthesize glycine impaired cell growth implying glycine dependent processes beyond an inability to synthesize heme can impact cell fitness. One of these downstream glycine dependent processes is folate synthesis. Interestingly, yeast that can synthesize folate *de novo* while higher eukaryotes including zebrafish and humans have a dietary requirement for folate. With this in mind, we sought to determine if the addition of exogenous glycine plus folate together could ameliorate the zebrafish model of CSA. When glycine and folate were added in combination to *slc25a38a+b* morphants, the level of hemoglobin was increased to 80% that observed in normal zebrafish (Fig 6B and 6C).

## Discussion

We determined that Hem25, the yeast homologue of human SLC25A38, is a mitochondrial glycine transporter that plays an important role in providing glycine for the first enzymatic step in heme synthesis, which is catalyzed in the mitochondria by Hem1 in yeast and ALAS2 in red blood cells and their precurors in humans. The function of Hem25 was determined through assessing phenotypes in yeast cells lacking Hem25 function where the import of glycine into mitochondria was required for yeast cells to grow and included the use of glycine as a sole nitogen source and glycine as a source of one carbon units. We also determined that Hem25 is not a mitochondrial serine importer using three lines of evidence. First, inactivation of gene encoding the mitochondrial enzyme that converts serine to glycine, *SHM1*, did not decrease heme levels; second, inactivation of the *SHM1* gene in cells lacking Shm2 function results in a growth defect due to a decreased capacity to synthesize one carbon units, while inactivation of the HEM25 gene in cells lacking Shm2 function did not decrease growth; and third, similarly to cells lacking Shm1, cells lacking the GCV also grow slowly in cells lacking Shm2 function due to an inability to synthesize one carbon units. Inactivation of *HEM25* in cells lacking both Shm2 and the GCV did not further decrease growth. Expression of human *SLC25A38* in yeast cells where the *HEM25* was gene deleted was able to restore heme to a normal level indicating conservation of function. We conclude that Hem25 and SLC25A38 are mitochondrial glycine importers. Future directions will be biochemical glycine transport experiments to determine substrate affinity, and whether SLC25A38 is a facilitative transporter or if there is a secondary active metabolite.

The conclusion that Hem25 and SLC25A38 are mitochondrial glycine importers was boltered by our work that determined the *de novo* source of glycine for heme synthesis in yeast. We found that Gly1, a cytosolic threonine aldolase, was the main source of glycine for heme synthesis, while the mitochondrial and cytosolic serine hydroxymethyltransferase, Shm1 and Shm2 respectively, did not significantly contribute to heme synthesis. A cytosolic source of *de novo* glycine as the main source of glycine for heme synthesis is consistent with the requirement for a high affinity glycine importer for subsequent synthesis of heme. As expected from this conclusion, loss of function of Gly1 along with Hem25 did not further decrease heme level as both are on a linear pathway for the synthesis of heme. Interestingly, loss of function of Gly1 along with Hem25 did result in a growth defect. This growth defect was partially restored by the addition of 5-Ala and completely restored by glycine supplementation. This indicates that the growth impairment of the double mutant is partly due to a decrease in heme level and partly due to second important role for glycine in the mitochondria such as the synthesis of one carbon units and/or the synthesis of serine. In line with this conclusion was our observation that there was no growth impairment in cells lacking only Hem25 function, and both glycine and 5-Ala restored heme level to normal in cells lacking Hem25.

We subsequently determined if glycine or 5-Ala could increase the level of hemoglobin in a more complex vertebrate model of CSA. We employed the zebrafish model as a substantial number of human blood disorders including inherited anemias have been accurately recapitulated in zebrafish [23,24]. Like many genes, *SLC25A38* is duplicated in the zebrafish genome thus translation-blocking morpholinos were designed to target *slc25a38a+slc25a38b* morphant embryos, which demonstrated reduced hemoglobin content. Surprisingly, the addition of glycine or 5-Ala at half of the maximum tolerated dose did not restore hemoglobin levels. In the case of 5-Ala, a higher dose may be required as 2 mM 5-Ala was found to rescue morphants where the expression of the protein required for PLP binding to *alas2* (*clpxa*) was decreased [25], whereas we used a dose of 0.3 mM as we observed some developmental abnormalities occuring with doses of 5-Ala above 1 mM. Another explanation is that *clpxa* may not provide as complete a block in 5-aminolevulinic acid synthase activity as compared to reducing the expression of *alas2* itself. In addition, 5-Ala was unable to restore growth to some yeast strain backgrounds lacking genes beyond *HEM25*, implying that glycine metabolism requirements for cells beyond heme synthesis can impact the capacity of 5-Ala to restore cell fitness. Surprisingly, the addition of glycine was also unable to increase the level of hemoglobin in *slc25a38a+b* morphants, especially as glycine restored heme and growth to yeast lacking *HEM25* and yeast strains lack *HEM25* in combination with impaired glycine metabolism.

The inability of glycine to increase hemoglobin levels in zebrafish prompted us to consider differences between yeast and vertebrate organisms that might impact glycine metabolism. A major difference is the ability of yeast to synthesize their own folate, whereas in zebrafish and higher eukaryotes folate is a vitamin that must be consumed in the diet. Mitochondria contain up to 40% of total cellular folate [26,27], and coupled with the fact that folate and its metabolites are normally found near the level required for use in folate dependent catalysis, a change in folate availability can have significant consequences [18]. It is also known that an inability to import folate into the mitochondria of Chinese hamster ovary cells results in a requirement for glycine for cell survival [28,29]. Finally, the CH_2_-THF dependent synthesis of glycine from serine by serine hydroxymethyltransferases has an essential role in one-carbon metabolism in mitochondria in mammalian cells [30–32]. We found that the addition of glycine plus folate substantially increased hemoglobin level in the zebrafish *SLC25A38* congenital sideroblastic anemia model. In the absence of *slc25a38a+b* function the ability to supply mitochondrial glycine becomes limiting. As *de novo* mitochondrial glycine synthesis requires folate derivatives, the addition of folate would increase mitochondrial glycine, providing glycine for the production of heme/hemoglobin (S6 Fig).

Human red blood cells rely primarily on the CH_2_-THF dependent serine hydroxymethyltransferases enzymes SHMT1 and SHMT2 for endogenous glycine synthesis [33,34], as the human threonine aldolase (*GLY1*) homologue is a non-functional pseudogene [35], and an alternate source of glycine found in many eukaryotes threonine dehydrogenase (including mice and zebrafish, but not yeast) is also a non-functional pseudogene in humans [36]. This implies an increased reliability on exogenous glycine and folate in humans. The substitution of medically, socially and financilly burdensome chronic transfusion therapy with commercially established and available glycine and folate is currently being explored as a potential innovative therapy for *SLC25A38* CSA patients on a clinical protocol that has been approved by our institutional research ethics board.

## Materials and Methods

### Ethics Statement

All zebrafish studies were approved by the Dalhousie University Committee on Laboratory Animals (Protocol #11–130) and conducting in accordance with the Canadian Council on Animal Care guidelines.

### Yeast Media and Culture Conditions

Yeast cells were maintained in YEPD (1% bacto-yeast extract, 2% bacto-peptone, 2% dextrose) medium or SD (0.67% bacto-yeast nitrogen base without amino acids, 2% dextrose) medium supplemented as required for plasmid maintenance and nutrient auxotrophies. Yeast cells were grown at 30°C.

### Yeast Strain and Plasmid Construction

The yeast strains used in this study are shown in S2 Table. Single deletion mutants in the BY4741 background were obtained from Euroscarf. The genetic KanMX6 marker was replaced with the nourseothricin acetyltransferase (Nat) gene or hygromycin resistance gene cassette using plasmid pAG25 or pAG32, respectively. Gene deletions were transfered from the BY4741 strains into the W303 background by PCR amplification of the corresponding selectable marker disrupted gene flanked at both ends by ~0.2 Kb of yeast genomic DNA, followed by transformation of W303 yeast cells and selection and characterization of transformants for the gene deletion by genomic PCR. Genomic PCR was used to verify all strain constructions. To screen for a putative second glycine importer among the members of the SLC25 family double gene deletion strains were constructed in the BY4741 background by standard genetic crosses of *hem25*Δ::NAT with the thirty one single gene deletion strains carrying mutations for each member of the yeast SLC25 family, followed by sporulation and haploid cell selection. Routinely, cells from the BY4741 background express a wild type allele of *HAP1* from a plasmid. The human *SLC25A38* open reading frame was PCR amplified from a B lymphocyte cDNA library and subcloned into the yeast expression vector p416-GPD. The DNA sequence of the *SLC25A38* open reading frame was confirmed by Sanger sequencing.

### Heme Determination

Logarithmically growing cells were harvested, washed in ice-cold water, and resuspended in 10 mM Tris-HCl, pH 8, 150 mM NaCl. Cells were lysed by glass bead beating for two periods of 1 min intercalated with 1 min on ice. Cell debris was removed by centrifugation at 500 × *g* for 3 min, and heme and protein content were assayed on the supernatants. Heme determination was carried out with the Hemin Assay Kit from BioVision using a Thermo Labsystems Multiskan Ascent Microplate Reader. Protein was determined by a modified Lowry method.

### Glycine Mass Determination

Free glycine content in yeast cells was estimated by the ninhydrin method. Briefly, yeast cells grown into log phase in SD media were harvested, washed with ice-cold water and resuspended at a density of 4 x10^8^ cells per ml of 10% sulphosalicylic acid containing 0.5 mM norleucine. Cells were broken by glass bead beating for 4 minutes and whole cell extracts were clarified by centrifugation at 18,000 x *g* for 30 min. Cell extracts were subjected to amino acid quantification using the Biochrom 30 Amino Acid Analyzer.

### Morpholino Injection

Casper zebrafish (*Danio rerio*) were raised and mated using routine procedures. Antisense morpholinos (MO) were ordered from Gene Tools. Antisense MOs targeted to the region immediately at the translational start site, with sequences 5′-CCGGATGAGCCACAGAGAACTCCAT-3′ for *slc25a38a* and 5′-CAGGATGAGCCAGGGCAACTTCCAT-3′ for *slc25a38b*. The 5-mismatch negative control morpholinos for *slc25a38a* were CCcGATGAcCCACAcAGAAaTCaAT MO and for *slc25a38b* were CAGcATGAcCCAGcGCAAaTTCaAT, the MO 5'-AACGAACGAACGAACGAACGAACGC-3’ was also used as an additional negative control. An antisense MO blocking the zebrafish *alas2*, 5′-CAGTGATGCAGAAAAGCAGACATGA-3′, was used as a positive control for phenotypes assocaited with decreased heme/hemoglobin synthesis. For each, 1.5 nl of MO was microinjected into casper embryos at the one-cell stage at a concentration of 0.5 mM, the maximum dose of MO that resulted in the least overt developmental abnormalities during the first 48 h of embryonic development. Attempts to detect *slc25a38a* or *slc25a38b* encoded protein using several commercially available antibodies were unsuccessful. In addition, we oberved that injection of mRNA coding for zebrafish *slc25a38a+b*, or human *SLC25A38*, at increasing doses resulted in either no phenotype or embryo death making rescue experiments impossible.

### Glycine, 5-Ala, and Folate Treatment of Zebrafish Embryos

Optimization of glycine and 5-Ala dosing was performed in wild type zebrafish embryos by toxicity studies evaluating the frequency of death and developmental abnormalities relative to the dose of drug, with 50% of the maximum tolerated dose chosen for treatments which were 100 mM for glycine and 1 nM for 5-Ala. At 24 hours postinjection (hpi) with MO, glycine or 5-Ala was added to egg water of the *slc25a38a+b*, *alas2*, and control morphants. Folic acid was converted to its sodium salt and added to fish water to give a final concentration of 1 mM at 6 hpf and then exchanged once at 24 hpf. The folate level used was that previously used to fully ameliorate selenite toxicity in zebrafish embryos [37]. Embryos were subsequently stained with *o*-dianisidine solution.

### *o*-Dianisidine Staining

At 48 hpi, embryos were decorionated and anesthetized with 0.02% Tricaine. The dechorionated embryos were stained in the dark for 15 min in *o*-dianisidine solution (0.6 mg/ml) containing 0.65% hydrogen peroxide, 40% ethanol, 10 mM sodium acetate, pH 4.5) for 15 min, fixed in 4% parafomaldehyde in phosphate buffered saline overnight at 4°C, and then embedded into 2.0% (w/v) low-melting point agarose for imaging.

### *In Situ* Hybridization

RNA in situ hybridization for zebrafish *slc25a38a* and *slc25a38b* genes was performed as previously described[38]. *In vitro* transcription was performed using the Roche DIG RNA labeling kit. Probes were generated from PCR products amplified directly from an oligo-dT primed 24 hpf zebrafish cDNA preparation using primers specific for *slc2538a* and *b*

### Fluorescence-Activated Cell Sorting (FACS) and Cell Collection

Transgenic *gata1*::*EGFP* embryos were dissociated to single cells as described previously[39] and then sorted using the FACS Aria I at the IWK Health Centre FACS Core Facility. Positive and negative sorted cells were put on ice immediately after collection, centrifuged and immediately processed for RNA extraction. The purity of sorted cell populations was verified by a repeated FACS analysis of sorted cells.

### Quantitative PCR (qPCR) Gene Expression Assays

RNA for qPCR experiments was extracted from 100,000 to 500,000 FACS-sorted cells, genomic DNA was removed, and cDNAs for qPCR assays were generated using 9-mer random primers and M-MuLV reverse in the presence of Protector RNAse Inhibitor. For SYBR Green I-based expression quantification we used the QuantiFast SYBR Green RT-PCR Kit (Qiagen). qPCR gene expression assays for *hbbe1*, *hbbe3*, *slc25a38a* and *slc25a38b* and 18S ribosomal RNA were performed.

## Supporting Information

S1 Fig Maximum-likelihood phylogenetic tree of human sequences in the SLC25 protein family of mitochondrial transporters. SLC25A38 is marked with an asterisk and sequences are annotated according to their transport roles. SLC25-family members were selected from Refseq based on previous analyses and aligned using HMMER and automatically edited using the AliMask-CS (Alignment Masking with Confidence Scores) algorithm. A maximum-likelihood phylogenetic tree was constructed using FastTree2 with the WAG substitution matrix and the resulting tree visualized with Figtree.
(PDF)

S2 Fig Hem25 deficiency does not affect *shm2*Δ *gcv1*Δdouble mutant growth. Cells of the indicated genotypes were grown in SD medium at 30°C. Growth was determined by optical density (OD) of the culture at 600_nm_. Data shown are the mean ± SEM for five independent segregants for *shm2*Δ *gcv1*Δ cells and six independent segregants for *shm2*Δ *gcv1*Δ *hem25*Δ cells.
(PDF)

S3 Fig Whole-mount *in situ* hybridizations with probes for *slc25a38a* and *slc25a38b* at 24 and 34 hpf stages of development demonstrate predominant erythroid expression. To confirm preferential expression in red blood cells, *EGFP*-positive erythrocytes were isolated by FACS from *gata1*:*EGFP* (where erythroid lineage cells are labeled by EGFP) transgenic zebrafish embryos. Quantitative PCR assays for *hbbe1*, *hbbe3*, *slc25a38a* and *slc25a38b* were performed on cDNA made from FACS-sorted positive and negative cells. Gene expression qPCR values were normalized to 18S ribosomal RNA values. Relative expression values to EGFP-positive cells are presented. Error bars indicate standard errors from quantitative PCR experiments done on 3 separate FACS-sorted cell samples. *** *p* < 0.001, ** *p* < 0.01, * *p* < 0.05 for the t-test between expression values for the EGFP-positive and negative cells.
(PDF)

S4 Fig Erythroid cells isolated from *slc25a38a+b* morphants are morphologically distinct but do not contain any pathological iron deposits compared to cells from control morphants. GFP positive cells were isolated by fluorescence activated cell sorting (FACS) from 48 hpf *gata1*:*EGFP* embryos, concentrated onto slides using standard cytospin protocols, and stained with Wright-Giemsa staining for gross morphology and erythroid cell identification (left) and Perls’ Prussian Blue for iron deposits (right). While no pathological iron deposits were detected in the *slc25a38a+b* morphant cells, these cells are larger with less compact nuclei, indicating a different level of maturation compared with control cells. Images are of randomly selected cells from the same experiment. The experiment was replicated three times with similar results.
(PDF)

S5 Fig 5-Aminolevulinic acid does not restore hemoglobin levels to *alas2* or *slc25a38a+b* morphants. Zebrafish embryos at 48-hpf were stained for hemoglobin with *o*-dianisidine following microinjection (at 1-cell stage) with the *alas2* or control standard MO. A) The typical phenotypes of control standard morpholino (STD MO) and *alas2* morpholino-injected embryos stained with *o*-dianisidine are shown. B) The morphants were incubated with or without 0.3 mM of 5-Ala added to the egg water at 4 hours post-injection. The level of *o*-dianisidine hemoglobin staining was scored visually as “low”, “medium” or “normal” in a blinded manner and the proportions of embryos in each category are presented in the graph. Results of three separate experiments are presented with the actual numbers of scored embryos indicated on the right hand axis of the graph. There was no statistically significant difference between untreated and 5-Ala-treated morphants indicating that 5-Ala supplementation did not increase hemoglobin level.
(PDF)

S6 Fig *De novo* mitochondrial glycine synthesise requires folate. Mitochondrial glycine synthesis requires folate derivatives for its synthesis from serine. In the absence of substantive glycine import due to mutation of the *SLC25A38* gene, mitochondria will require higher *de novo* synthesis of glycine to supply glycine for subsequent synthesis of heme/hemoglobin. In vertebrates such as zebrafish and humans folate is a vitamin that must be taken up in the diet and can become limiting, whereas the *yeast S*. *cerevisiae* has the capacity to synthesize folate.
(PDF)

S1 Table Toxicity curves for 5-Ala and glycine for zebrafish embryos. (DOCX)

S2 Table *S*. *cerevisiae* strains used in this study. (DOCX)
