# Supplementary figures and images for: Glycine and Folate Ameliorate Models of Congenital Sideroblastic Anemia
Source: PLoS Genet. 2016 Jan 28;12(1):e1005783. doi: 10.1371/journal.pgen.1005783 (PMC4731144; doi:10.1371/journal.pgen.1005783)

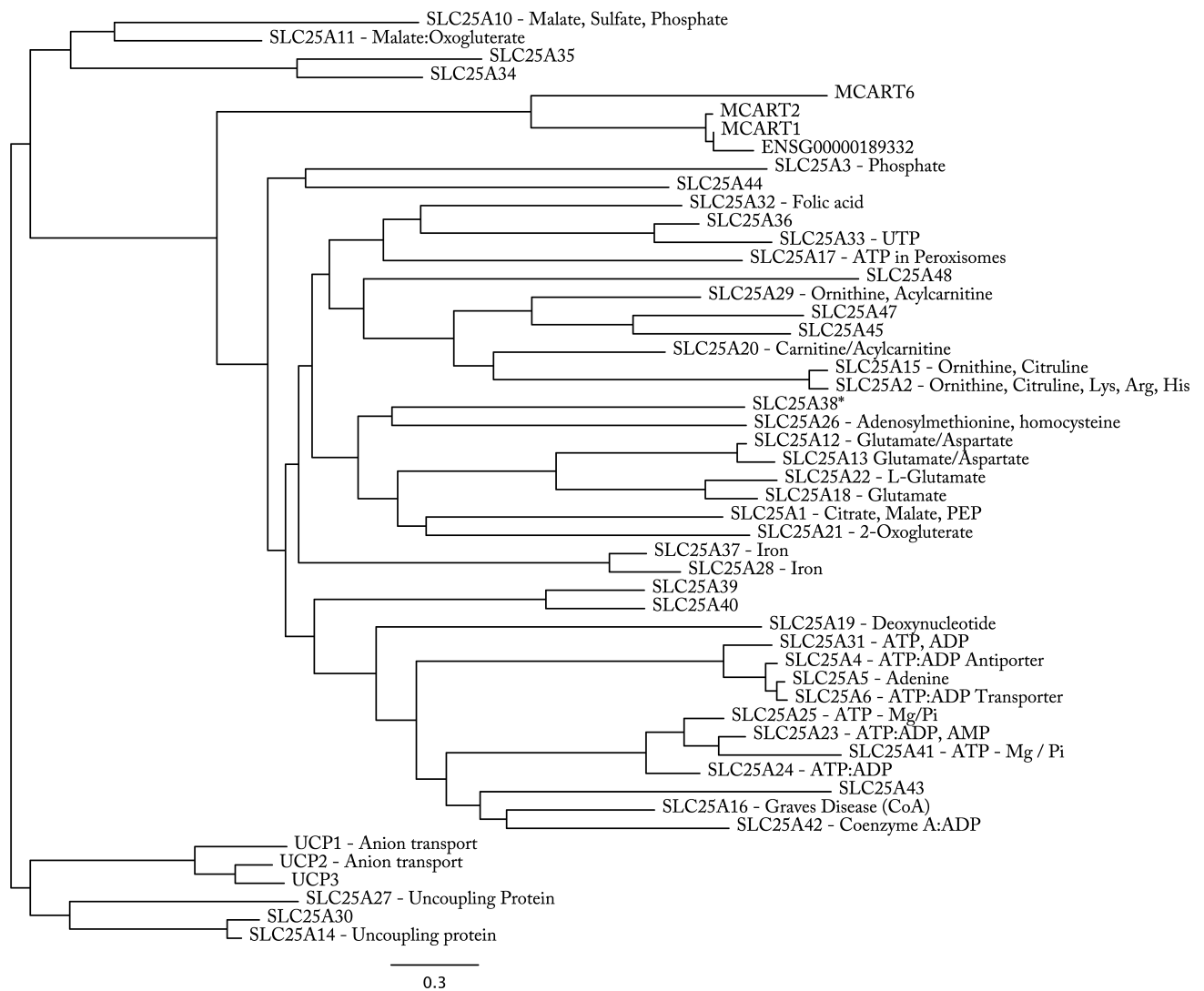

Supplementary Figure 1

Supplement: S1 Fig — SLC25A38 is marked with an asterisk and sequences are annotated according to their transport roles. SLC25-family members were selected from Refseq based on previous analyses and aligned using HMMER and automatically edited using the AliMask-CS (Alignment Masking with Confidence Scores) algorithm. A maximum-likelihood phylogenetic tree was constructed using FastTree2 with the WAG substitution matrix and the resulting tree visualized with Figtree. (PDF) [file pgen.1005783.s001.pdf]

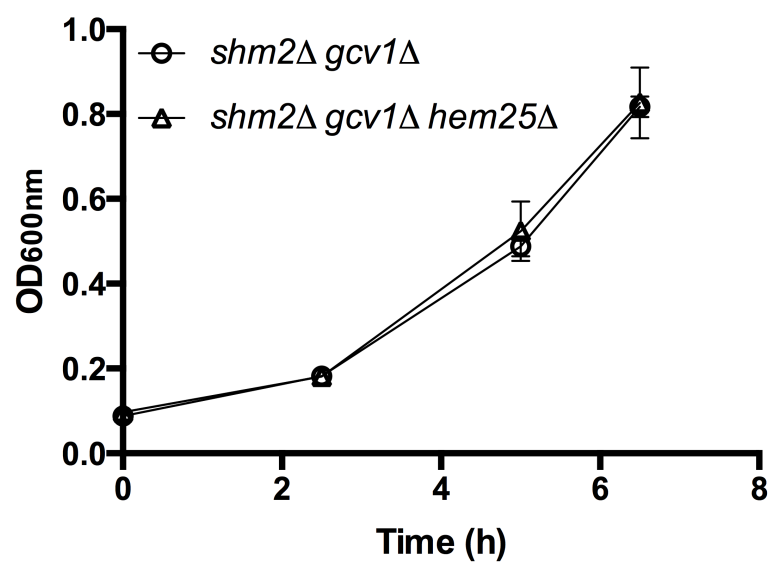

Supplementary Figure 2

Supplement: S2 Fig — Cells of the indicated genotypes were grown in SD medium at 30°C. Growth was determined by optical density (OD) of the culture at 600nm. Data shown are the mean ± SEM for five independent segregants for shm2Δ gcv1Δ cells and six independent segregants for shm2Δ gcv1Δ hem25Δ cells. (PDF) [file pgen.1005783.s002.pdf]

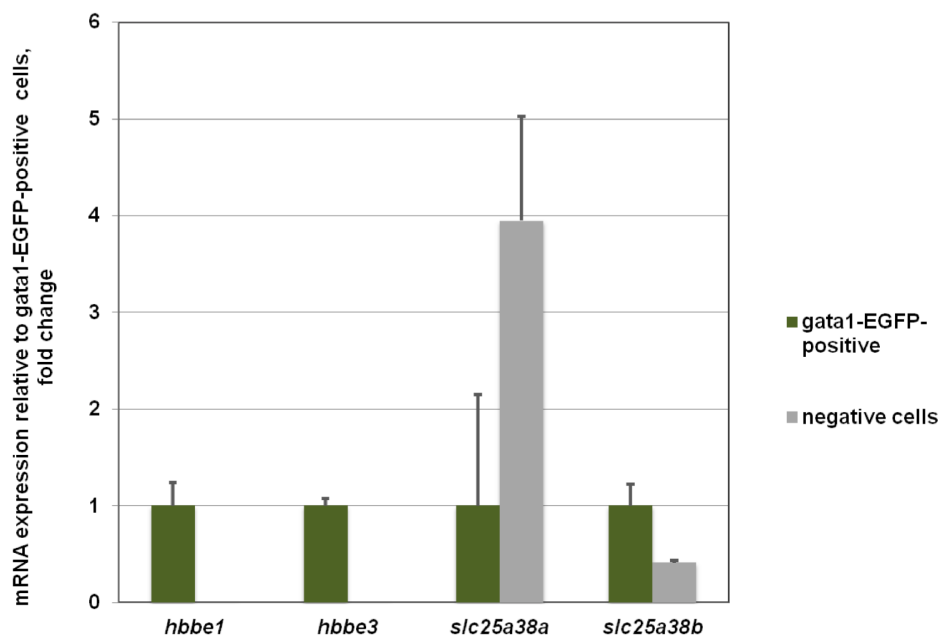

Supplementary Figure 3

Supplement: S3 Fig — To confirm preferential expression in red blood cells, EGFP-positive erythrocytes were isolated by FACS from gata1:EGFP (where erythroid lineage cells are labeled by EGFP) transgenic zebrafish embryos. Quantitative PCR assays for hbbe1, hbbe3, slc25a38a and slc25a38b were performed on cDNA made from FACS-sorted positive and negative cells. Gene expression qPCR values were normalized to 18S ribosomal RNA values. Relative expression values to EGFP-positive cells are presented. Error bars indicate standard errors from quantitative PCR experiments done on 3 separate FACS-sorted cell samples. *** p < 0.001, ** p < 0.01, * p < 0.05 for the t-test between expression values for the EGFP-positive and negative cells. (PDF) [file pgen.1005783.s003.pdf]

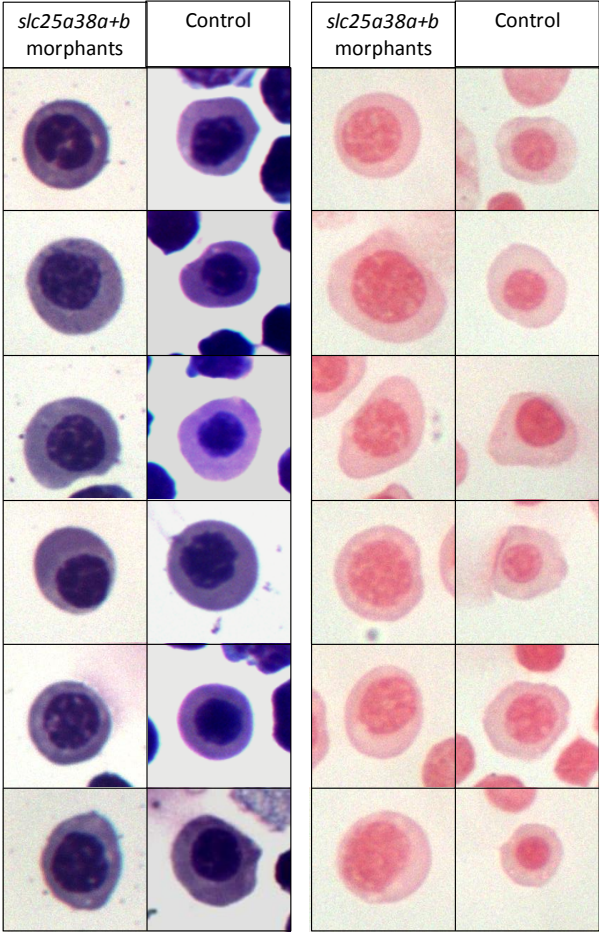

Supplementary Figure 4

Supplement: S4 Fig — GFP positive cells were isolated by fluorescence activated cell sorting (FACS) from 48 hpf gata1:EGFP embryos, concentrated onto slides using standard cytospin protocols, and stained with Wright-Giemsa staining for gross morphology and erythroid cell identification (left) and Perls’ Prussian Blue for iron deposits (right). While no pathological iron deposits were detected in the slc25a38a+b morphant cells, these cells are larger with less compact nuclei, indicating a different level of maturation compared with control cells. Images are of randomly selected cells from the same experiment. The experiment was replicated three times with similar results. (PDF) [file pgen.1005783.s004.pdf]

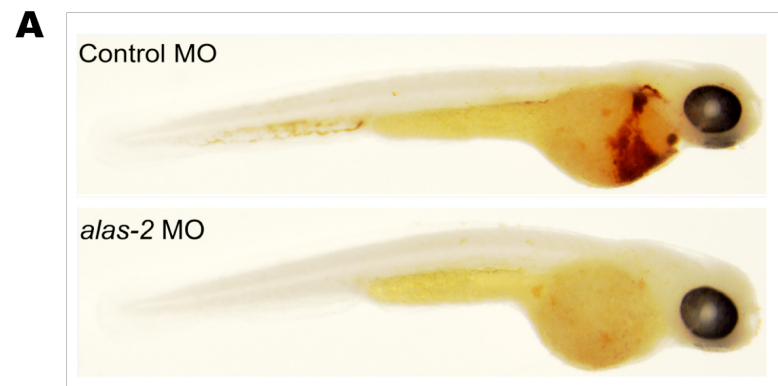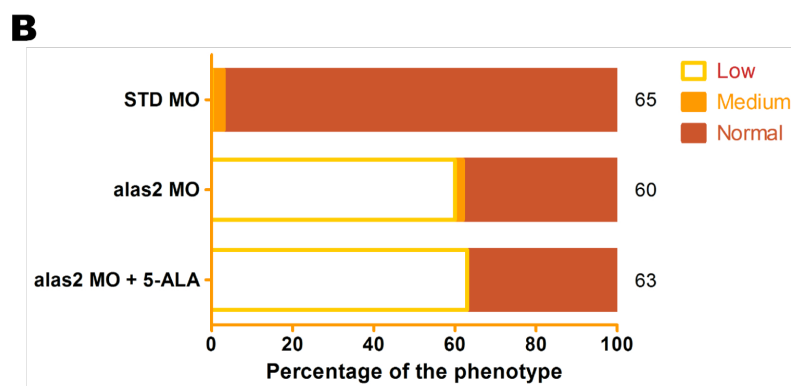

Supplementary Figure 5

Supplement: S5 Fig — Zebrafish embryos at 48-hpf were stained for hemoglobin with o-dianisidine following microinjection (at 1-cell stage) with the alas2 or control standard MO. A) The typical phenotypes of control standard morpholino (STD MO) and alas2 morpholino-injected embryos stained with o-dianisidine are shown. B) The morphants were incubated with or without 0.3 mM of 5-Ala added to the egg water at 4 hours post-injection. The level of o-dianisidine hemoglobin staining was scored visually as “low”, “medium” or “normal” in a blinded manner and the proportions of embryos in each category are presented in the graph. Results of three separate experiments are presented with the actual numbers of scored embryos indicated on the right hand axis of the graph. There was no statistically significant difference between untreated and 5-Ala-treated morphants indicating that 5-Ala supplementation did not increase hemoglobin level. (PDF) [file pgen.1005783.s005.pdf]

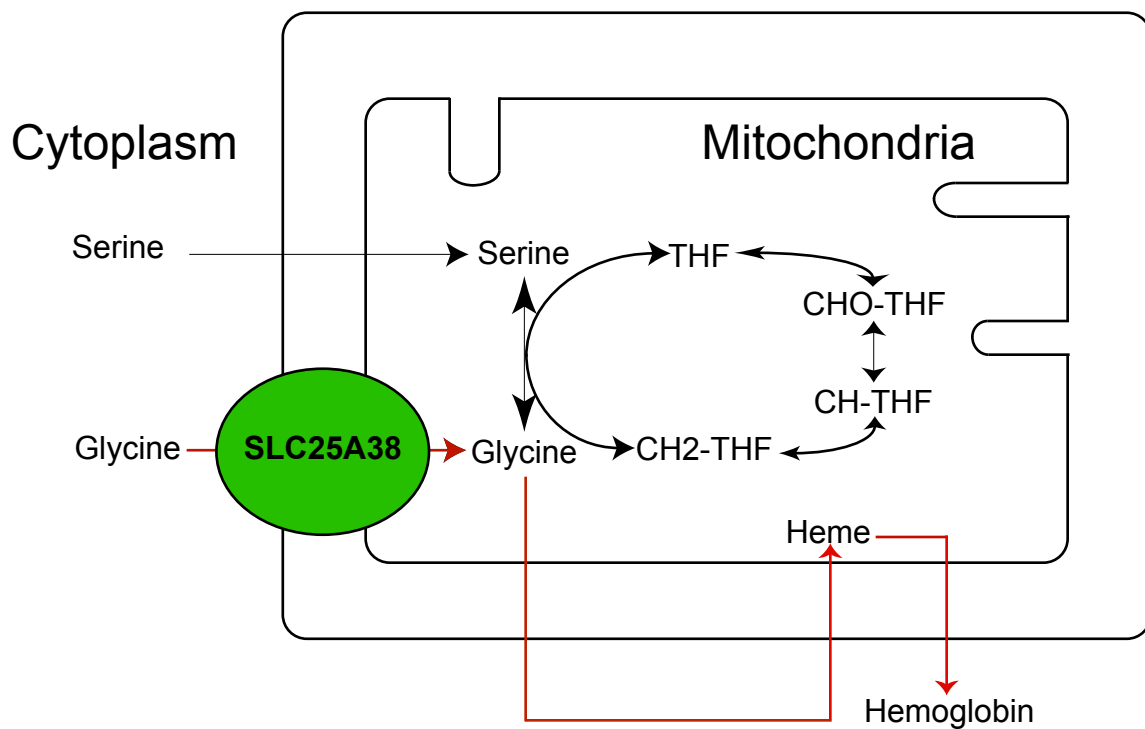

Supplementary Figure 6

Supplement: S6 Fig — Mitochondrial glycine synthesis requires folate derivatives for its synthesis from serine. In the absence of substantive glycine import due to mutation of the SLC25A38 gene, mitochondria will require higher de novo synthesis of glycine to supply glycine for subsequent synthesis of heme/hemoglobin. In vertebrates such as zebrafish and humans folate is a vitamin that must be taken up in the diet and can become limiting, whereas the yeast S. cerevisiae has the capacity to synthesize folate. (PDF) [file pgen.1005783.s006.pdf]
