# Supplementary material for: Glycine and Folate Ameliorate Models of Congenital Sideroblastic Anemia
Source: PLoS Genet. 2016 Jan 28;12(1):e1005783. doi: 10.1371/journal.pgen.1005783 (PMC4731144; doi:10.1371/journal.pgen.1005783)
Supplement: S1 Table — (DOCX) [file pgen.1005783.s007.docx]

**S1 Table. Toxicity curves for 5-Ala and glycine for zebrafish embryos.**

| **Chemical** | **Concentration, mM** | **Survival at 48-hpf treatment** | **Total** | **Percentage survival** |
| --- | --- | --- | --- | --- |
| 5-Ala | 0 | 73 | 104 | 70.2 |
| 5-Ala | 0.3 | 65 | 98 | 66.3 |
| 5-Ala | 1 | 77 | 112 | 68.8 |
| 5-Ala | 2 | 14 | 108 | 13.0 |
| 5-Ala | 3 | 0 | 108 | 0.0 |
| glycine | 0 | 60 | 60 | 100.0 |
| glycine | 50 | 60 | 60 | 100.0 |
| glycine | 100 | 58 | 60 | 96.7 |
| glycine | 200 | 52 | 60 | 86.7 |
| glycine | 400 | 24 | 60 | 40.0 |
