# Supplementary material for: Glycine and Folate Ameliorate Models of Congenital Sideroblastic Anemia
Source: PLoS Genet. 2016 Jan 28;12(1):e1005783. doi: 10.1371/journal.pgen.1005783 (PMC4731144; doi:10.1371/journal.pgen.1005783)
Supplement: S2 Table — (DOCX) [file pgen.1005783.s008.docx]

**S2 Table. *S. cerevisiae* strains used in this study.**

| ***Strain*** | ***Genotype*** |
| --- | --- |
| NDY 046 | W303 *hem25*Δ::NAT |
| NDY 323 | W303 *shm1*Δ::KanMx |
| NDY 252 | W303 *shm2*Δ::KanMx |
| NDY 254 | W303 *gly1*Δ::KanMx |
| NDY 086 | W303 *hem1*Δ::NAT |
| NDY 263-265 | W303 *hem25*Δ::NAT *gly1*Δ::KanMx |
| NDY 387 | W303 *ser1*Δ::Hyg |
| NDY 432 | W303 *ser1*Δ::Hyg *hem25*Δ::NAT |
| NDY 466 | W303 *lpd1*Δ::KanMx |
| NDY 261 | W303 *shm2*Δ::KanMx  *hem25*Δ::NAT |
| NDY 608-612 | W303 *gcv1*Δ::NAT *shm2*Δ::Hyg |
| NDY 613-618 | W303 *gcv1*Δ::NAT *shm2*Δ::Hyg  *hem25*Δ:: KanMx |
| NDY 003 | Y2454 *hem25*Δ::NAT [pRS416-*HAP1*] |
| NDY 229 | Y2454 *hem25*Δ::NAT *ymc1*Δ::KanMx [pRS416-*HAP1*] |
